# End of life care preferences among people of advanced age: LiLACS NZ

**DOI:** 10.1186/s12904-017-0258-0

**Published:** 2017-12-19

**Authors:** Merryn Gott, Rosemary Frey, Janine Wiles, Anna Rolleston, Ruth Teh, Tess Moeke-Maxwell, Ngaire Kerse

**Affiliations:** School of Nursing, Faculty of Medical and Health Sciences, University of Auckland, Auckland, New Zealand

**Keywords:** Culture, End of life, Preferences, Māori, Indigenous, Palliative, Advanced age, Older people, Ethnicity

## Abstract

**Background:**

Understanding end of life preferences amongst the oldest old is crucial to informing appropriate palliative and end of life care internationally. However, little has been reported in the academic literature about the end of life preferences of people in advanced age, particularly the preferences of indigenous older people, including New Zealand Māori.

**Methods:**

Data on end of life preferences were gathered from 147 Māori (aged >80 years) and 291 non- Māori aged (>85 years), during three waves of Te Puawaitangi O Nga Tapuwae Kia Ora Tonu, Life and Living in Advanced Age (LiLACs NZ). An interviewer-led questionnaire using standardised tools and including Māori specific subsections was used.

**Results:**

The top priority for both Māori and non-Māori participants at end of life was ‘not being a burden to my family’. Interestingly, a home death was not a high priority for either group. End of life preferences differed by gender, however these differences were culturally contingent. More female Māori participants wanted spiritual practices at end of life than male Māori participants. More male non-Māori participants wanted to be resuscitated than female non- Māori participants.

**Conclusions:**

That a home death was not in the top three end of life priorities for our participants is not consistent with palliative care policy in most developed countries where place of death, and particularly home death, is a central concern. Conversely our participants’ top concern – namely not being a burden – has received little research or policy attention. Our results also indicate a need to pay attention to diversity in end of life preferences amongst people of advanced age, as well as the socio-cultural context within which preferences are formulated.

## Background

Palliative care policy internationally now prioritises service provision which is in line with how both individuals, and wider societies, define ‘good dying’ [1]. Unsurprisingly, therefore, there is now a growing body of research examining preferences for end of life care. However, key gaps in current knowledge and understanding exist regarding end of life care preferences. In particular, little is known regarding the preferences of people of ‘advanced age’, defined within the gerontology literature as those aged >85 years [2, 3]. This knowledge gap is limiting the development of policy appropriate to the needs of ageing populations internationally [4] because, not only are the numbers of deaths in the oldest age group rising rapidly in most developed countries [5], but also the limited research in this area indicates some unique aspects of care preferences within this group [6–8].

There has also been a lack of attention to diversity in preferences amongst the oldest old; for example, explorations of the intersection of age and culture on preferences for end of life care are rare. In particular we are not aware of any research that has explicitly explored end of life preferences amongst indigenous people of advanced age. Within the New Zealand (NZ) context, the lack of attention paid to the unique aspirations and needs of indigenous Māori is highly problematic given that ‘partnership, protection and participation’ of Māori is enshrined in law. Māori are less likely to use specialist palliative care services than NZ Europeans, which may partly reflect a lack of cultural appropriateness of these services [9, 10], but little research has explored the unique preferences of Māori for end of life care. It is within this context that this paper reports research exploring the end of life (EOL) preferences of Māori >80 years and non-Māori >85 years of age. A differential recruitment age was adopted because disparities in the burden of chronic disease, reflected in a 7 year difference in life expectancy [11, 12], mean that Māori are considered to reach ‘advanced age’ earlier than non-Māori.

### Research aim

To explore the end of life care preferences of Māori and non-Māori of advanced age and identify associations between preferences and key socio-demographic factors.

### Research objectives

1. To describe the EOL preferences of Māori and non-Māori participants.
2. To examine the relationship between EOL care preferences and key socio-demographic factors (gender and culture).

## Methods

### Study background

Te Puawaitanga o Nga Tapuwae Kia Ora Tonu, Life and Living in Advanced Age: a Cohort Study in New Zealand (LiLACS NZ), was set up under joint Māori and non-Māori leadership to identify predictors of successful ageing, defined in relation to quality of life, health status, functional independence and longevity. Ethical approval for the study was granted by the Northern X Regional Ethics Committee of New Zealand. This paper reports findings from a sub-study of LiLACS NZ which focused specifically upon exploring the End of Life (EOL) preferences of Māori and non-Māori.

### Recruitment

Participants were recruited in 2010 from the Bay of Plenty and northern part of the Lakes areas of New Zealand, regions chosen as they contain a proportionately high number of Māori. In total 1636 eligible participants (766 Māori and 870 non-Māori) were identified via the NZ General and Māori electoral rolls (*n* = 1564) (in NZ adults are required by law to register for these rolls), from general practices (*n* = 46), by word of mouth (*n* = 22), from residential care (*n* = 3) and by advertisement (n = 1). Of these, 937 participants were enrolled in LiLACS NZ: 421 participants were Māori (55% of total eligible) and 516 were non-Māori (59% of total eligible). The formal criteria for eligibility for LiLACS NZ were non-Māori born between 1 January and 31 December 1925 (aged 85 in 2010), and Māori born between 1 January 1920 and 31 December 1930 (aged 80 to 90 in 2010). Those meeting the age criteria needed to have been living within the recruitment areas during the 2010 enrolment year. Using 2006 census data with projections to 2010 the sex and age distribution of Māori participants was consistent with the general population; for non-Māori, more men than would be expected were recruited (LiLACS NZ 48% vs census 42%) [13]. An extensive community consultation process identified local and tribal bodies to conduct the study fieldwork; participants were followed up across six yearly administrations (waves). Participants undertook either a full questionnaire or six pages of core questions. Predictors of attrition included: death, ill-health and loss of interest [7]. The questionnaires were administered face-to-face by trained interviewers offered as home visits or at another site chosen by the participant.

## Questionnaire

A full description of the questionnaire contents is provided elsewhere [14]. Culturally specific questions were developed by the researchers, a te RoopuKaitiaki o nga tikanga Māori [caregiver/guidance] Group of Māori elders and drew on the Te Whāre Tapa Whā model of health [15]. The questionnaire was translated into Te Reo Māori (Māori language) appropriate to the region of study. Pertinent to the current study the following measures were included:

### Socio-demographic questions

Socio-demographic characteristics collected (Wave 1) included age, gender and marital status as well as length of time widowed, separated or divorced. Level of education achieved and standard of living questions were drawn from 9the New Zealand Census (2006) and Health Work and Retirement Study/New Zealand and the Longitudinal Study of Ageing (NZLSA) questionnaire [16] respectively. Family relationship questions (e.g. brothers, sisters, sons and daughters) were adapted from the Newcastle 85+ study protocols [17].

### End of life preferences

EOL questions were introduced in Wave 3 with the End of Life (EOL) tool. The questionnaire drew on questions ranking the importance of various factors to a ‘good death’ developed by Waghorn, Young and Davies (2011), and the VOICES questionnaire [18, 19]. Participants first answered the question: “Are you comfortable talking about your plan for the end of life?” (yes/no) Participants who agreed then answered seven questions related to their EOL planning in a dichotomous (yes/no) format. This section included questions related to Living Will or Advanced Care Plans (e.g. Enduring Power of Attorney, resuscitation status, medical intervention status), whether they had spoken to a relative regarding their wishes and whether or not there would be spiritual practices performed while dying. Participants were also asked to rate the importance of twelve end-of-life spiritual, cultural and practical end of life preferences (e.g. to have pain/symptoms controlled, to be at peace with God) on a scale from 1 “not important” to 5 “extremely important.” [14].

## Analysis

Quantitative data were coded into SPSS version 22. Both descriptive (frequencies, percentages) and inferential statistics appropriate to the level of measurement (Chi-square *x* ^*2*^) were employed. The five-point EOL preferences items were collapsed into three categories: 1 “not important”, 2 (combined 2 + 3) “moderately important”, and 3 “very important” (combined 4 + 5). Small sample sizes required the collapsing of categories to obtain the recommended expected value of five cases per contingency table cell for chi-square analyses [20]. Ranking of importance of each preference item represents the valid percent reporting the item as “very important”. Valid percent was utilised to adjust for differences in total number of responses on each preference item, thereby allowing for comparison between items. Education and marital status variables were analysed as dichotomous categories, (no school qualification/school qualification) and (married/not married).

### Presentation of results

In terms of results presentation, the principle of ‘equal explanatory power’ supports the inclusion of equal numbers of Māori and non-Māori participants in all health research [21]. Adopting this principle demands separate analyses of data for Māori and non-Māori with equal power [22]. Thus side by side comparisons should not be conducted. As stated by Nazroo [21] side by side comparisons could contribute to the ‘racialisation’ of health issues “by identifying the health disadvantage of ethnic minority groups as inherent to their ethnicity, a consequence of their cultural and genetic ‘weaknesses’ rather than a result of the disadvantage they face because of the ways in which their ethnicity or race is perceived by others.” (p.215) We also are cognisant of the differing age criteria for the two groups, complicating comparative analyses.

## Results

In Wave One 421 persons identified as Māori, while 516 identified as non- Māori. In Wave 3, 438 (147 Māori and 291 non-Māori) participants responded “yes” to a question asking whether they were comfortable with discussing EOL issues (Table 1) and completed the seven end of life planning questions, representing 76% of total Wave 3 Māori participants and 86% of total Wave 3 non- Māori participants [22].

**Table 1:** Overview of EOL participant socio-demographic characteristics frequency and percent (*n* = 438)

	Māori (147)		Non-Māori (291)	
	Frequency	Percent	Frequency	Percent
Gender				
*Male*	57	38.8	147	50.5
*Female*	90	61.2	144	49.5
Age				
*82 to 86*	102	69.4	0	0
*87 to 92*	45	30.6	291	100
Ethnicity (multiple response)				
*European*	66	44.9	264	90.7
*Samoan*	1	0.7	0	0
*Chinese*	1	0.7	0	0
*Indian*	0	0	0	0
*Other European*	12	8.2	32	11
*Other*	1	0.7	5	1.7
Marital Status				
*Never Married/Partnered*	3	2	9	3.1
*Married/Partnered*	51	34.7	108	37.1
*Widow/widower*	85	57.8	164	56.4
*Separated*	3	2	1	0.3
*Divorced*	5	3.4	8	2.7
Education				
*Primary School or No School*	39	26.5	45	15.5
*Secondary School no qualification*	59	40.1	107	36.8
*Secondary School*	25	17	59	20.3
*Trade/Occupational*	7	4.8	32	11
*Tertiary Qualification*	14	9.5	47	16.2
Who do you live with most of the time?				
*Alone*	54	36.7	150	51.5
*Spouse/partner only*	41	27.9	104	35.7
*Spouse and child/other relative*	10	6.8	8	2.7
*Spouse and non-relatives*	1	0.7	1	0.3
*Child not spouse*	26	17.7	12	4.1
*Others (not spouse or child)*	13	8.8	15	5.2
What best describes your home?				
*Private dwelling*	113	76.9	159	54.6
*Private unit or apartment*	15	10.2	46	15.8
*Unit or apartment –family*	4	2.7	4	1.4
*Retirement village –own villa*	6	4.1	61	21
*Rest Home*	4	2.7	9	3.1
*Private Hospital*	1	0.7	4	1.4
*Marae or Iwi based housing*	1	0.7	0	0
*Other*	3	2	7	2.4
Support Services (e.g. meal service, cleaning shopping or personal care assistance)				
*Yes*	55	38.7	146	53.7
*No*	87	61.3	126	46.3

### Wave 3 sample representativeness

According to Kerse et al. [22] compared to Wave 1, Wave 3 participants were more likely to: have been independent in activities of daily living (ADLs); be less deprived [New Zealand Deprivation Index score (NZdep)[19]; had fewer health conditions; and had a decreased likelihood of depression at baseline than those who did not complete Wave 3. Please see Kerse et al. [22] for further cohort comparisons.

#### EOL respondents

Utilising chi-square test of independence, there were no significant differences (*p* > .05) between the participants who answered yes to the comfort in discussing EOL question and those who did not (*n* = 21) based on gender X^2^(1, *n* = 459) =8.44, *p* = .004; ethnicity Māori (y/n) X^2^(1, n = 459) = .184, *p* = .668; marital status (married/not married) X^2^(1, n = 459) =6.31, *p* = .012; education (education/no education) X^2^(1, *n* = 454) =0.39, *p* = .534, or living circumstances (alone/not alone) X^2^(1, *n* = 456) = 0.84, *p* = .358.

### Socio-demographic characteristics

The socio-demographic characteristics of the participants who answered “yes” to the question related to comfort in discussing end-of-life plans and preferences are listed in Table 1.

#### Māori

Over half of the participants were female (61.2%) most often (69.4%) under the age of 87. Participants were most often widows/widowers (57.8%) and lived alone (10.8%). Home was most often described as a private dwelling (36.7%) and participants most often reported not utilising Support Services (e.g. cleaning, shopping or personal care assistance) (61.3%).

#### Non-Māori

Non-Māori participants were also predominantly female (49.5%) and over the age of 87 (100%). Like Māori participants, non-Māori participants were most often (56.4%) widows/widowers and lived alone (51.5%). Additionally, private dwelling was most often (54.6%) selected for home description. Slightly more participants reported using Support Services (53.7%) than not using Support Services (46.3%).

### Relative importance of end of life care preference items

The frequency of responses for each of the twelve EOL preferences addresses Objective One. Frequency of selection of “very important” was interpreted as a rough measure of the relative importance of the preference for Māori and non-Māori participants. These results are outlined below:

#### Māori

**Fig. 1:**
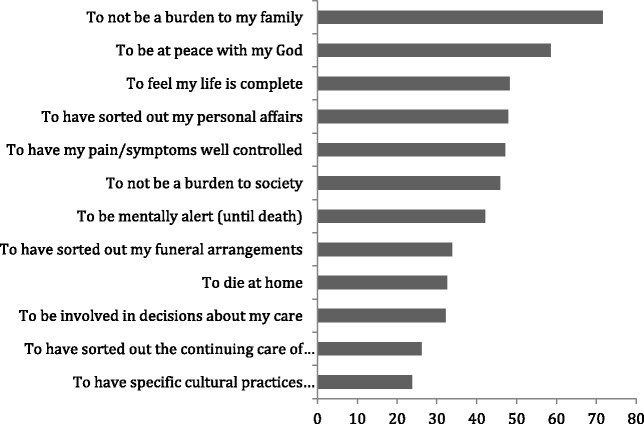
Māori End-Of Life Preferences Valid Percent of Responses (*n* = 147)

The most frequently reported EOL preference based on valid percent of the rating “very important” was “to not be a burden to family” (71.5%). Second in terms of valid percentage of responses was “to be at peace with my God” (58.3%) followed by “to feel my life is complete” (48.6%) (Fig. 1).

#### Non-Māori

**Fig. 2:**
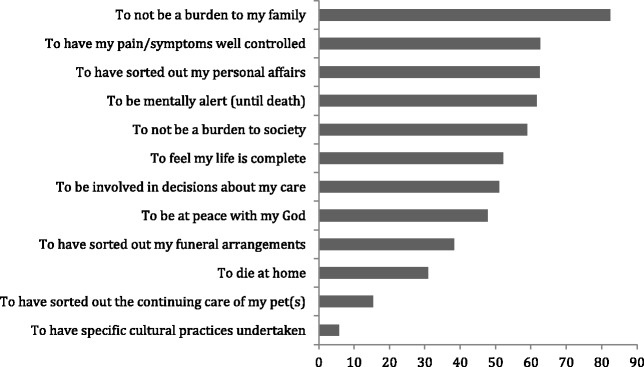
Non-Māori End-Of Life Preferences Valid Percent of Responses (*n* = 291)

Non-Māori participants likewise most frequently reported valid response was “to not be a burden to family” (82.6%), followed by “to have my pain and symptoms controlled” (62.3%) and “to sort out my personal affairs” (62.5%) (Fig. 2).

### End of life care and gender and culture

#### Plans

In order to address Objective Two, chi square tests were performed to examine differences in EOL planning and preferences question responses based on culture (Māori/non-Māori) and gender:

#### Māori

More women than men had talked to a family member about EOL wishes X^2^(1, *n* = 147) = 6.05, *p* = .014 and more women than men reported planning for spiritual practices at the EOL X^2^(1, *n* = 139) =6.85, *p* = .009, (Table 2).

**Table 2:** Māori end of life care planning frequency of response by gender (Chi Square statistic) (n = 147)

	Male		Female			
	Yes	No	Yes	No	X^2^	p
Do you have a Living Will or Advanced Care Plan?	26	28	40	50	0.19	0.667
Do you have an Enduring Power of Attorney?	23	5	38	11	0.23	0.635
Do you wish for major surgery or medical intervention if you are seriously ill?	10	11	13	27	1.32	0.251
Do you wish to be resuscitated?	10	15	11	31	1.37	0.242
Have you talked to a friend or family member about your wishes?	35	22	72	18	6.05	0.014*
Will there be spiritual practices while you are dying?	25	30	57	27	6.85	0.009*

X^2^ = chi square

*p < .05

#### Non-Māori

More men than women reported a wish to have major surgery or medical intervention X^2^ (1, *n* = 126) =8.22, *p* = .004. Similarly, more men than women reported a desire to be resuscitated X^2^(1, *n* = 130) = 4.26, *p* = .039. More women than men reported a preference for spiritual practices at EOL X^2^(1, *n* = 273) =6.11, *p* = .013, (Table 3).

**Table 3:** Non- Māori end of life care planning frequency of response by gender (Chi Square statistic) (*n* = 291)

	Male		Female			
	Yes	No	Yes	No	X^2^	p
Do you have a Living Will or Advanced Care Plan?	71	74	78	65	0.90	0.344
Do you have an Enduring Power of Attorney?	66	4	74	7	0.47	0.491
Do you wish for major surgery or medical intervention if you are seriously ill?	22	35	11	58	8.22	0.004*
Do you wish to be resuscitated?	13	44	7	66	4.26	0.039*
Have you talked to a friend or family member about your wishes?	107	39	110	34	0.37	0.544
Will there be spiritual practices while you are dying?	30	109	47	87	6.11	0.013*

* *p* < .05

### Preferences

#### Māori

Gender differences in responses to the 12 EOL preferences questions indicated that more women than men had a preference “to be at peace with God” X^2^(2, *n* = 144) = 9.77, *p* = .008. There were no other significant differences in EOL preferences based on gender (*p* > .05), (Table 4).

**Table 4:** Importance of EOL preferences for Māori frequency and percent () by gender (Chi Square statistic) (*n* = 147)

	Male			Female				
	Not at all	Moderately	Very	Not at all	Moderately	Very	X^2^	*p*
a. To have my pain/symptoms well controlled	4 (7.4)	27 (50.0)	23 (42.6)	8 (9.4)	34 (40.0)	43 (50.6)	1.35	0.509
b. To not be a burden to my family	3 (5.6)	14 (25.9)	37 (68.5)	5 (5.6)	19 (21.1)	66 (73.3)	0.45	0.798
c. To feel my life is complete	1 (1.9)	28 (52.8)	24 (45.3)	3 (3.4)	41 (46.1)	45 (50.6)	0.76	0.683
d. To be at peace with my God	11 (20.0)	18 (32.7)	26 (47.3)	4 (4.5)	27 (30.3)	58 (65.2)	9.77	0.008*
e. To have sorted out my personal affairs	5 (9.3)	29 (53.7)	20 (37.0)	3 (3.4)	38 (42.7)	48 (53.9)	4.97	0.083
f. To die at home	10 (18.9)	27 (50.9)	16 (30.2)	16 (17.8)	43 (47.8)	31 (34.4)	0.27	0.872
g. To be mentally alert (until death)	1 (1.9)	28 (52.8)	24 (45.3)	11 (12.8)	40 (46.5)	35 (40.7)	4.95	0.084
h. To be involved in decisions about my care	4 (7.6)	33 (62.3)	16 (30.2)	9 (10.1)	50 (56.2)	30 (33.7)	0.58	0.750
i. To have sorted out my funeral arrangements	9 (16.4)	27 (49.1)	19 (34.6)	15 (16.9)	45 (50.6)	29 (32.6)	0.06	0.971
j. To not be a burden to society	7 (13.0)	20 (37.0)	27 (50.0)	7 (8.1)	42 (48.3)	38 (43.7)	2.06	0.358
k. To have specific cultural practices undertaken	22 (41.5)	15 (28.3)	16 (30.2)	36 (40.5)	35 (39.3)	18 (20.2)	2.53	0.282
l. To have sorted out the continuing care of my pet(s)	20 (52.6)	10 (26.3)	8 (21.1)	34 (50.0)	15 (22.1)	19 (27.9)	0.67	0.714

X^2^ Chi Square statistic

*p < .05

#### Non-Māori

Chi-square tests of independence were performed to examine the relationship between gender and the list of 12 EOL preferences. Women more often than men rated pain and symptom control X^2^(2, *n* = 276) = 13.65, *p* = .001; to feel my life is complete X^2^(2, *n* = 276) = 11.86, *p* = .003; to be at peace with God X^2^(2, *n* = 276) = 24.11, *p* < .0001; to have sorted out my personal affairs X^2^(2, *n* = 288) = 10.38, *p* = .006; to be mentally alert, X^2^(2, *n* = 282) = 12.24, *p* = .002; to be involved in decisions X^2^(2, *n* = 284) = 17.59, *p* = .0002; and not to be a burden to society X^2^(2, *n* = 288) = 18.52, *p* < .0001 as “very important”, (Table 5).

**Table 5:** Importance of EOL preferences for non-Māori frequency and percent () by gender (Chi Square Statistic) (*N* = 291)

	Male			Female				
	Not at all	Moderately	Very	Not at all	Moderately	Very	X^2^	*p*
a. To have my pain/symptoms well controlled	11 (8.1)	55 (40.4)	70 (51.5)	5 (3.6)	33 (23.6)	102 (72.9)	13.65	0.001*
b. To not be a burden to my family	3 (2.1)	27 (18.8)	114 (79.2)	4 (2.8)	16 (11.1)	124 (86.1)	3.38	0.185
c. To feel my life is complete	11 (7.9)	69 (49.6)	59 (42.5)	5 (3.7)	46 (33.6)	86 (62.8)	11.86	0.003
d. To be at peace with my God	43 (30.7)	49 (35)	48 (34.3)	16 (11.8)	36 (26.5)	84 (61.8)	24.11	<.0001**
e. To have sorted out my personal affairs	3 (2.1)	64 (44.4)	77 (53.5)	3 (2.1)	38 (26.4)	103 (71.5)	10.38	0.006*
f. To die at home	35 (25.2)	66 (47.5)	38 (27.3)	34 (25.4)	54 (40.3)	46 (34.3)	1.89	0.390
g. To be mentally alert (until death)	5 (3.6)	61 (43.6)	74 (52.9)	8 (5.6)	34 (23.9)	100 (70.4)	12.24	0.002*
h. To be involved in decisions about my care	10 (7.1)	76 (53.9)	55 (39)	10 (7.0)	43 (30.1)	90 (62.9)	17.59	0.0002*
i. To have sorted out my funeral arrangements	31 (21.5)	66 (45.8)	47 (32.6)	26 (18.1)	54 (37.5)	64 (44.4)	4.24	0.120
j. To not be a burden to society	3 (2.1)	70 (48.6)	71 (49.3)	9 (6.3)	36 (25.0)	99 (68.8)	18.52	<.0001**
k. To have specific cultural practices undertaken	96 (69.1)	34 (24.5)	9 (6.5)	100 (75.2)	26 (19.6)	7 (5.3)	1.27	0.531
l. To have sorted out the continuing care of my pet(s)	74 (74.0)	13 (13.0)	13 (13.0)	60 (67.4)	13 (14.6)	16 (18.0)	1.14	0.567

X^2^ Chi Square statistic

*ns* (33.3%) of cells have expected count less than 5

*p < .05, **p < .001

## Discussion

Understanding end of life priorities is a critical element of ensuring high quality palliative and end of life care. This study provides new information regarding the end of life priorities of people in advanced age, amongst whom demand for palliative care is predicted to increase exponentially in coming decades. In particular, we have shown that priorities for care differ from published data regarding the priorities of young old people who are typically in good health [23], and that there are also significant differences in end of life care priorities by key socio-demographic factors and notably, amongst Māori and non-Māori participants and men and women.

Our findings also highlight the importance of considering the end of life preferences of older people within the context of the pervasive ageism experienced in many countries internationally and the low societal value placed on old age [24]. For example, both Māori and non-Māori participants ranked ‘not being a burden’ as their top priority at end of life. This confirms previous qualitative studies with older people [4, 25–27], as well as quantitative research with ‘terminally ill’ people of all ages, of whom 19–65% are estimated to experience some form of self-perceived burden [28]. That such concerns may be particularly marked amongst older people is unsurprising given evidence that the negative societal framing of ageing populations has fed into older people’s perceptions that they are ‘a burden’ [29]. In addition, previous research has indicated that the perception of being a burden to others is a factor that underlies many requests for assisted dying among patients with life-limiting illnesses [30–32]. This current findings have important practice and policy implications in relation to debates surrounding this issue [33].

These findings also lend weight to the argument that older people’s end of life preferences are not well explained by the Western individualistic model of bioethics [34, 35] that underpins much palliative care policy and practice, but rather fit better with a more relational ‘ethics of care’ model [36] which is based on the social connectedness, and “total care” of a person [37]. For older Māori it is likely that their aroha (love, care, compassion) acts to protect whānau already burdened with economic hardship from being further encumbered with the high financial costs associated with end of life care [38].

It is also important to highlight that a home death did not feature in the top three end of life care priorities for either Māori or non-Māori participants. Such findings lend further weight to the need to revisit normative understandings that a ‘good death’ must occur at home [39], understandings which permeate palliative care policy internationally (for example, [40–42]. Our participants identified that pain and symptom control, attending to spiritual concerns (for Māori) and ‘getting affairs in order’ (for non-Māori) were more important than the actual place of dying. These latter two concerns are rarely given full consideration in policy or practice [43], with spirituality, in particular, often poorly attended to [44]. In New Zealand, in line with the principle of active protection of Te Tiriti o Waitangi (Treaty of Waitangi), there is an urgent need to consider ways to ensure spiritual requirements are understood and respected so that Māori dying in advanced age are able to exert *tino rangatiratanga* (self-determination) in their end of life journey.

A recent review drew attention to the failure of palliative care research to adequately address the influence of gender on end of life circumstances [45]. This study further confirms that this influence is pervasive, but culturally contingent, as the effect of gender on end of life priorities differed for Māori and non-Māori participants. For example, female Māori participants were more likely to prioritise spiritual practices at end of life than male Māori participants. Māori women were also more likely to have talked with family about end of life preferences than had men. This may reflect the culturally specific tribal role of Māori women to attend their ill and dying [46]. While gender may play a role in the reported differences, for Māori the source of difference is largely informed by culture, whakapapa (genealogical/relational connections to the ill and dying) and *wairuatanga* (spirituality) [47].

The influence of gender on end of life care preferences and priorities was even more marked for non-Māori participants, with more men than women expressing a preference for medical intervention and resuscitation. Earlier research has also indicated less desire for life-sustaining treatments for women than men [48, 49]. One possible explanation is the lower proportion of older women who have available family and whānau to care for them when compared to men [50], but findings might also point to the potential impact of both ageism and sexism upon the ways in which older women make end of life decisions. End of life preferences for NZ Europeans appear to be highly gendered, a finding with important practical implications for practice and policy and which deserves far greater exploration given the lack of gender analyses conducted in palliative care research to date [45].

For Māori, cultural practices provide both spiritual and physical connections. These connections with whānau, the land, ancestors and with a force greater than self, provide both strength and comfort for the journey to come [44]. Tikanga (cultural customs, values, and practices) “serve as markers of identity and more importantly are about connecting whānau members with the person dying, as well as with each other” [51] (p. 48). This need for connection is reflected in the reported EOL planning questions related to speaking to a family member about EOL wishes.

### Limitations

Whilst these data make a unique contribution to existing knowledge and understanding, certain limitations must be acknowledged. Firstly, there was attrition over the five years of the LiLACS NZ study with the loss of more Māori than non-Māori (10%–12%) [7]. The availability of alternative sources of data for outcomes mitigates this limitation to some extent [7]. For those participants offered the opportunity to respond to the end-of-life questions in Wave 3, non-completion was related to participant comfort in discussing end of life concerns. This resulted in reduced numbers of responses to some items (e.g. number of Māori respondents to ‘Planning’ questions) which may impact on the generalizability of these results. However, no significant differences in demographic characteristics between responders and non-responders were identified. This finding is also interesting in and of itself as it supports previous research indicating that some older people prefer to adopt a ‘closed awareness’ attitude towards death [52] and do not welcome a discussion of end of life preferences. Rather, not talking about dying and living ‘day-to-day’ represents a coping strategy [53], something which is important to remember given policy drives in many countries to increase end of life discussions, for example through Advance Care Planning (e.g. [40]. Indeed, this stance does not fit well with policy directives that regard such a discussion as a quality indicator of good end of life care (e.g. [40, 41].

Use of self-report questionnaire with Māori participants may be subject to cultural variations in response style. Individuals of different cultural backgrounds may use answer response scales in different ways [54]. A more qualitative approach that takes into account culturally specific requirements may yield greater depth of understanding of Māori EOL preferences [55]. There is an obvious wairua [spiritual] connection when a Māori person talks about death and dying. Research by MacDonald (2016) found that participants who were more connected to ‘being Māori’ tended to have a greater sense or awareness of their spirituality [56]. This may have also influenced willingness to discuss end of life preferences. Other demographic variables in addition to gender and culture may also have contributed to differences in plans and preferences noted in this study. For example, Māori health literacy scores have been reported to be lower than non- Māori [57] which may affect choices and preferences for care at the end-of-life. [58, 59].

## Conclusion

Overall, findings highlight the need to attend to not only the nature of end of life preferences for people in advanced age, but also the socio-cultural context within which these are formulated. This context has not previously been investigated in any detail. However, our findings indicate the need for more research, particularly in relation to the ways in which cultural identity and gender shape end of life care preferences. Such research is crucial to achieving the goal of meeting end of life preferences at end of life for all people of advanced age.

## Abbreviations

EOL: End of Life
NZ: New Zealand

## References

[1] World Health Organisation: Palliative Care. Fact Sheet No 402. http://www.who.int/mediacentre/factsheets/fs402/en/. Accessed 18 Apr 2016.

[2] Smith J, Borchelt M, Maier H, Jopp D (2002). Health and well–being in the young old and oldest old. J Soc Issues.

[3] Gwozdz W, Sousa-Poza A (2010). Ageing, health and life satisfaction of the oldest old: an analysis for Germany. Soc Indic Res.

[4] Fleming J, Farquhar M, Brayne C, Barclay S (2016). Death and the oldest old: attitudes and preferences for end-of-life care - qualitative research within a population-based cohort study. PLoS One.

[5] Gott M, Ingleton C (2011). How can we improve palliative care provision for older people? Global perspectives. BMJ Support Palliat Care.

[6] Hunt RW, Fazekas BS, Luke CG, Roder DM (2001). Where patients with cancer die in South Australia, 1990-1999: a population-based review. Med J Aust.

[7] Kerse N, Teh R, Moyes S, Broad J, Rolleston A, Gott M, Kepa M, Wham C, Hayman K, Jatrana S (2015). Cohort profile: Te puawaitanga o Nga tapuwae Kia Ora tonu, life and living in advanced age: a cohort study in New Zealand (LiLACS NZ). Int J Epidemiol.

[8] Cohen J, Gott M, Van den Block L, Albers G, Pereira S, Onwuteaka-Philepsen B, Pasman O, Deliens L (2015). Dying in place in old age: public health challenges. Palliative care for older people: public health perspective.

[9] Kidd J, Reid S, Collins N, Gibbons V, Black S, Blundell R, Peni T, Ahu H (2014). Kia Mau te Kahu Whakamauru: health literacy in palliative care.

[10] Ministry of Health (New Zealand) (2014). Palliative care and Māori from a health literacy perspective.

[11] Yon Y, Crimmins E (2014). Cohort morbidity hypothesis: health inequalities of older Māori and non-Māori in New Zealand. N Z Popul Rev.

[12] Statistics New Zealand. New Zealand Period Life Tables:2012–2014. https://www.stats.govt.nz/information-releases/new-zealand-abridged-period-life-table-201416-final. Accessed 20 June 2017.

[13] Dyall L, Kepa M, Hayman K, Teh R, Moyes S, Broad J, Kerse N (2013). Engagement and recruitment of Māori and non-Māori people of advanced age to LiLACS NZ. Aust NZ J Public Health.

[14] Hayman KJ, Kerse N, Dyall L, Kepa M, Teh R, Wham C (2012). Life and living in advanced age: a cohort study in New Zealand-Te Puāwaitanga o Nga Tapuwae Kia Ora Tonu, LiLACS NZ: study protocol. BMC Geriatr.

[15] Durie MH (1985). A Māori perspective of health. Soc Sci Med.

[16] Stevenson B, Waldegrave C, King P, Alpass F, Stephens C, Towers A, et al. New Zealand longitudinal studies of ageing. https://www.massey.ac.nz/massey/fms/Colleges/College%20of%20Humanities%20and%20Social%20Sciences/Psychology/HART/publications/NZLSA_%20Research-Summary_2014.pdf?234C87488161797F7A0665730159566E. Accessed 21 June 2017.

[17] Collerton J, Barrass K, Bond J, Eccles M, Jagger C, James O, Martin-Ruiz C, Robinson L, von Zglinicki T, Kirkwood T (2007). The Newcastle 85+ study: biological, clinical and psychosocial factors associated with healthy ageing: study protocol. BMC Geriatr.

[18] Waghorn M, Young H, Davies A (2011). Opinions of patients with cancer on the relative importance of place of death in the context of a ‘good death’. BMJ Support Palliat Care.

[19] Addington-Hall J, McCarthy M (1995). Regional study of Care for the Dying: methods and sample characteristics. Palliat Med.

[20] McHugh ML (2013). The chi-square test of independence. Biochem Med.

[21] Nazroo JY, Dorling D, Simpson L (1998). The racialisation of ethnic inequalities in health. Statistics in society: the arithmetic of politics.

[22] Kerse N, Teh R, Moyes SA, Broad J, Rolleston A, Gott M, Kepa M, Wham C, Hayman K, Jatrana S (2015). Cohort profile: Te Puawaitanga o Nga Tapuwae Kia Ora Tonu, life and living in advanced age: a cohort study in New Zealand (LiLACS NZ). Int J Epidemio.

[23] Carr D, Khodyakov D (2007). End-of-life health care planning among young-old adults: an assessment of psychosocial influences. J Gerontol B Psychol Sci Soc Sci..

[24] Angus J, Reeve P (2006). Ageism: a threat to “aging well” in the 21st century. J Appl Gerontol.

[25] Gott M, Small N, Barnes S. S P, et al. Older people's views of a good death in heart failure: implications for palliative care provision. Soc Sci Med. 2008;67:1113–21. 10.1016/j.socscimed.2008.05.024 18585838

[26] Gott M, Seymour J, Bellamy G, Clark D, Ahmedzai S (2004). Older people's views about home as a place of care at the end of life. Palliat Med.

[27] Seymour J, Gott M, Bellamy G, Ahmedzai SH, Clark D (2004). Planning for the end of life:the views of older people about advance care statements. Soc Sci Med.

[28] McPherson CJ, Wilson KG, Murray MA (2007). Feeling like a burden to others: a systematic review focusing on the end of life. Palliat Med.

[29] Press Association. Older people ‘feel a burden’ to society. In: AgeUk. Uk: Press Association; 2013. http://www.ageuk.org.uk/latest-news/archive/older-people-feel-a-burden-to-society/. Accessed 4 May 2016.

[30] Coyle N, Sculco L (2004). Expressed desire for hastened death in seven patients living with advanced cancer: a phenomenologic inquiry. Oncol Nurs Forum.

[31] Kelly B, Burnett P, Pelusi D, Badger S, Varghese F, Robertson M (2002). Terminally ill cancer patients' wish to hasten death. Palliat Med.

[32] Rodríguez-Prat A, Balaguer A, Booth A, Monforte-Royo C. Understanding patients’ experiences of the wish to hasten death: an updated and expanded systematic review and meta-ethnography. BMJ Open. 2017;7(9):e016659. 10.1136/bmjopen-2017-016659 PMC5640102 28965095

[33] Kutner JS (2010). An 86-year-old woman with cardiac cachexia contemplating the end of her life: review of hospice care. JAMA.

[34] Beauchamp TL, Childress JF (2001). Principles of biomedical ethics.

[35] Gellie A, Mills A, Levinson M, Stephenson G, Flynn E (2015). Death: a foe to be conquered? Questioning the paradigm. Age Ageing.

[36] Noddings N (2013). Caring: a relational approach to ethics and moral education.

[37] Frey R, Powell L, Gott M (2013). Care vs. care: 'Biomedical' and 'Holistic' worldviews of palliative care. Eur J Integr Med.

[38] Gardiner C, Brereton L, Frey R, Wilkinson-Meyers L, Gott M (2014). Exploring the financial impact of caring for family members receiving palliative and end-of-life care: a systematic review of the literature. Palliat Med.

[39] Pollock K. Is home always the best and preferred place of death? BMJ. 2015; 351:h4855. (Online), 351 doi:10.1136/bmj.h4855. 10.1136/bmj.h4855 26446163

[40] Department of Health (UK) (2008). End of life care strategy: promoting high quality care for all adults at the end of life.

[41] Ministry of Health (New Zealand) (2001). The New Zealand palliative care strategy.

[42] Robinson J, Gott M, Gardiner C, Ingleton C (2016). The ‘problematisation’ of palliative care in hospital: an exploratory review of international palliative care policy in five countries. BMC Palliat Care.

[43] Steinhauser K, Christakis N, Clipp E, McNeilly M, McIntyre L, Tulsky J (2000). Factors considered important at the end of life by patients, family, physicians, and other care providers. JAMA.

[44] Duggleby W, Kuchera S, MacCleod R, Holyoke P, Scott T, Holtslander L (2015). Indigenous people's experiences at the end of life. Palliat Support Care.

[45] Morgan T, Williams LA, Trussardi G, Gott M (2016). Gender and family caregiving at the end-of-life in the context of old age a systematic review. Palliat Med.

[46] Beaglehole E, Beaglehole P (1945). Contemporary Māori death customs. J Polyn Soc.

[47] Lanyon-Orgill P. The origin of the oceanic languages. J Polyn Soc. 1943;52(2):25-45. Retrieved from http://www.jstor.org/stable/20702932.

[48] Bookwala J, Coppola KM, Fagerlin A, Ditto PH, Danks JH, Smucker WD (2001). Gender differences in older adults' preferences for life-sustaining medical treatments and end-of-life values. Death Stud.

[49] Carmel S, Mutran E (1997). Preferences for different life-sustaining treatments among elderly persons in Israel. J Gerontol B Psychol Sci Soc Sci.

[50] Roth DL, Haley WE, Wadley VG, Clay OJ, Howard G (2007). Race and gender differences in perceived caregiver availability for community-dwelling middle-aged and older adults. Gerontologist.

[51] Rauawaawa Kaumātua Charitable Trust (2012). Māori health literacy and communication in palliative care: Kaumātua-led models.

[52] Richards N, Ingleton C, Gardiner C, Gott M (2013). Awareness contexts revisited: indeterminacy in initiating discussions at the end-of-life. J Adv Nurs.

[53] Gott M, Ingleton C (2011). Living with ageing and dying: palliative and end of life care for older people.

[54] Smith PB (2004). Acquiescent response bias as an aspect of cultural communication style. J Cross Cult Psych.

[55] Gott M, Moeke-Maxwell T, Williams L, Black S, Trussardi G, Wiles J, et al. Te Pākeketanga: living and dying in advanced age--a study protocol. BMC Palliat Care. 2015;14:74. 10.1186/s12904-015-0073-4 PMC4687083 26691519

[56] MacDonald M (2016). Prevalence and aetiology of anaemia in older Māori and non-Māori.Doctoral dissertation.

[57] Ministry of Health (New Zealand) (2010). Korero Marama: health literacy and Māori results from the 2006 adult literacy and life skills survey.

[58] Rauawaawa Kaumātua Charitable Trust Research Project Team (2012). Māori health literacy and communication in palliative care: Kaumātua-led models.

[59] Volandes AE, Paasche-Orlow M, Gillick MR, Cook EF, Shaykevich S, Abbo ED, Lehmann L (2008). Health literacy not race predicts end-of-life care preferences. J Palliat Med.

